# Supplementary material for: Exosomal Vimentin from Adipocyte Progenitors Protects Fibroblasts against Osmotic Stress and Inhibits Apoptosis to Enhance Wound Healing
Source: Int J Mol Sci. 2021 Apr 28;22(9):4678. doi: 10.3390/ijms22094678 (PMC8125065; doi:10.3390/ijms22094678)
Supplement: Supplementary file 1 [file ijms-22-04678-s001.zip › ijms-1188351-supplementary.pdf]

Supplementary Figure S1

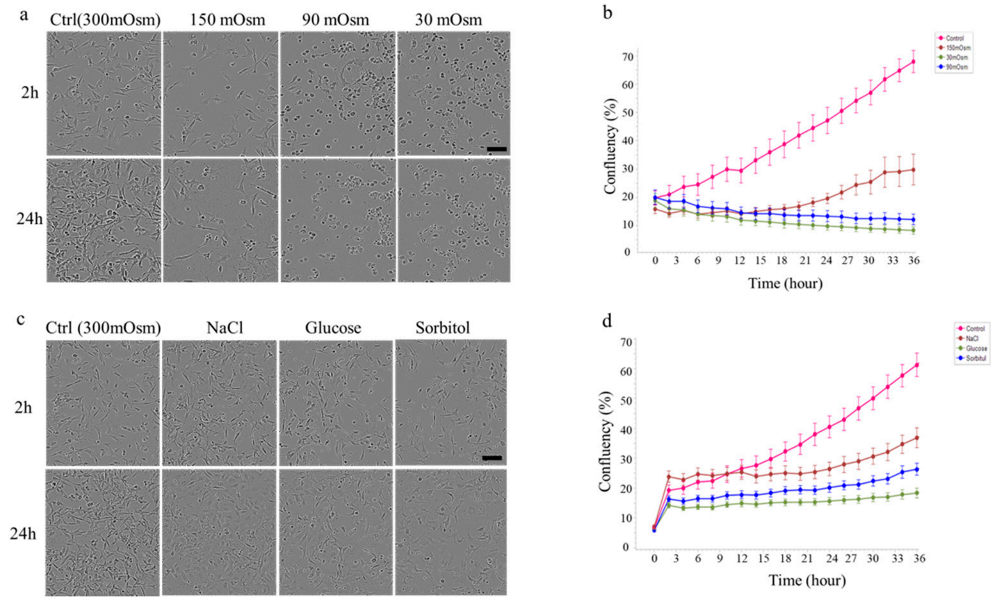

Figure S1. Hypo and Hyper-osmotic stress media optimization.(**a**) Representative images and (**b**) cell confluency of WT-APCs incubated in different hypotonic media following ratios: 1:9, 1:4, and 1:1 to obtain 30 mOsm, 60 mOsm, and 150 mOsm, respectively after 24 h. (**c**) Representative images and (**d**) cell confluency of WT-APCs incubated in different hypertonic media with the addition of 100 mM NaCl, 200 mM sorbitol, or 200 mM glucose for an increase of 200 mOsm kg^{-1} H2O after 24 h. Normal media (300mOsm) were used as control. Scale bar: 100 µm. * $p < 0.05$, ** $p < 0.01$, *** $p < 0.00$.
